# TOPS: an internet-based system to prevent healthy subjects from over-volunteering for clinical trials

**DOI:** 10.1007/s00228-012-1231-8

**Published:** 2012-02-17

**Authors:** M. Boyce, M. Walther, H. Nentwich, J. Kirk, S. Smith, S. Warrington

**Affiliations:** Hammersmith Medicines Research (HMR), Cumberland Avenue, London, NW10 7EW UK

**Keywords:** TOPS, Over-volunteering, Phase 1 trials, Healthy volunteers

## Abstract

**Aim:**

Our aim was to set up a system to help UK clinical research units to prevent healthy volunteers from participating in more than one non-therapeutic trial simultaneously, or from starting a second trial too soon after the first.

**Methods:**

TOPS (The Over-volunteering Prevention System) is internet-based, simple and quick to use, free to users and a charity run by a Board of Trustees. Users enter only two or three pieces of information: (1) ‘National Insurance number’ (NINO) of UK citizens, or ‘passport number’ and country of origin of non-UK citizens, as their identifier, (2) ‘date of last dose’ of trial medicine or (3) ‘never dosed’. Subjects must consent, but TOPS collects only non-personal data, so it does not require Ethics Committee approval and is not covered by the Data Protection Act.

**Results:**

A total of 55 research units (29 clinical research organisations, 5 pharmaceutical companies, 13 universities and 8 hospitals) throughout the UK have registered to use TOPS, and have entered 124,906 volunteers since we launched it. All commercial and many non-commercial units now use TOPS. In our unit, no subject has to the best of our knowledge participated in two trials simultaneously. TOPS has reduced to <1% the incidence of subjects attempting to volunteer within 3 months of completing another trial elsewhere, and very few have to our knowledge succeeded.

**Conclusion:**

TOPS is widely used and effective, and helps research units to comply with UK clinical trial regulations.

## Introduction

Healthy volunteers who take part in clinical trials are usually paid for their time and inconvenience, so there has always been concern that payment might tempt some to take part in trials too frequently and become ‘professional volunteers’ [1], or ‘over-volunteers’ [2].

There are anecdotal reports of healthy subjects over-volunteering, but no published data. Furthermore, there is inconsistent guidance on the minimum time between two trials. The Association of the British Pharmaceutical Industry (ABPI) Guidelines [2] recommend 3 months, and longer for a radioactive molecule or one with a long half-life; in contrast, the USA Food & Drug Administration (FDA) Guidelines [3] recommend 28 days.

During 1997–2001, we screened 6,998 healthy volunteers for trials in our unit: 68 (0.97%; 58 men and 10 women) had completed a trial elsewhere within the previous 3 months [4]. The average interval was 6 weeks, but for some subjects it was as short as 2 weeks. We detected over-volunteering by (1) calling other units when we noticed recent forearm venepuncture marks, remnants of electrocardiogram skin electrodes still attached or a microcytic blood film; (2) other units calling us for the same reasons; (3) learning from replies to our letters to the subjects’ general practitioner (GP). The units involved were contract research organisations (CROs), pharmaceutical companies and universities, in different parts of the UK.

Healthy subjects rarely derive therapeutic benefit from taking part in a clinical trial, so the risk of harm must be minimal [2]. Anyone who takes part in two trials simultaneously, or even takes part in a trial too soon after a previous one, is at increased risk. Therefore, we decided to set up TOPS (The Over-volunteering Prevention System).

We thought that for such a system to be used widely by both commercial and non-commercial units, it must: be simple, quick to use, internet-based, secure, and reliable; protect the anonymity of the subject; reveal no information of commercial interest; carry no advertising; be free to all types of user; be low-cost to set up and run.

## Methods

### Design

An ideal system would completely identify volunteers and track all studies that they undertake. We considered the subject’s name, photograph, fingerprint [5] and iris [6] as ideal identifiers, but rejected them all because:

- users would have to buy a software licence and recognition equipment for fingerprint or iris recognition, and
- making available a volunteer’s private details to all users is unreasonable and would require ethics committee approval and compliance with the Data Protection Act [7].

Therefore, to protect the volunteer’s privacy, TOPS stores minimal information:

- National Insurance number (NINO) of UK citizens, or passport number and country of origin of non-UK citizens, as a unique identifier, and
- date of last dose of trial medicine, or that the volunteer was never dosed.

Everyone who works or claims benefits in the UK must have a NINO, which is unique and permanent for each person [8]. The UK government gave us permission to use NINO.

To encourage wide use, TOPS is: internet-based; simple and quick to use; secure; free from information of commercial interest; validated according to Good Automated Manufacturing Practice [9]; free to all users. The TOPS database is stored in SQL Server. Users access the database indirectly through a web browser. The website https://www.tops.org.uk uses https to identify itself and to encrypt all data transferred across the internet.

Our aim was to avoid TOPS being linked to our unit, lest that deter other commercial units from using it. We failed to find an independent organisation to sponsor and run TOPS, so we registered it as a charity with a Board of Trustees [10], and we run it ourselves. Most of the members of the Board of Trustees are independent of our unit.

Since 2000, the cost of setting up and running TOPS, excluding our time, has been £25,995 (€30,154), almost all of which has been for the website host.

### Using TOPS

Potential users request (enquiries@TOPS.org.uk) a username and password. Contact details (name, phone number and email address) of each organisation are stored to facilitate contact among users.

Users must obtain the volunteer’s permission to store information about them. TOPS records the current date as the registration date and notifies the user of any previous entry for that volunteer, the date of the last dose (or that they were never dosed) and the unit concerned. If there is no previous entry or there is an acceptable interval since the last dose (or they were never dosed), the user proceeds to screen the volunteer. If there is a previous entry that suggests over-volunteering, the user contacts the unit that made the last entry. If over-volunteering is confirmed, the user rejects the volunteer.

When a user enters the date of the last dose, there is a box to tick to warn other users that the study is an unusually long one, such as one with a monoclonal antibody or one involving radioactivity.

We define over-volunteering as a subject attending for screening within 3 months of completing a previous study. TOPS does not distinguish between volunteers who received active treatment and those who received placebo in a previous study because it is usually impossible to obtain this information within 3 months after the study.

The number of trials that a subject may take part in during any 12-month period will depend on the circumstances (Table 1).

**Table 1:** Guidance on the use of healthy volunteers^a^

The number of trials that a subject may take part in during any 12-month period will depend on the:
• Types of investigational medicinal product (IMP) and their half-lives
• Routes of administration of the IMP
• Frequency and duration of exposure to IMP
• Procedures involved
• Total volume of blood taken from the subject
• Amount of radioactivity (no more than 10 milliSievert).
In general, subjects should not receive an IMP systemically less than 3 months after the previous one

^a^From Association of the British Pharmaceutical Industry (ABPI) guidelines for phase 1 clinical trials, 2007 [2]

## Results

**Fig. 1:**
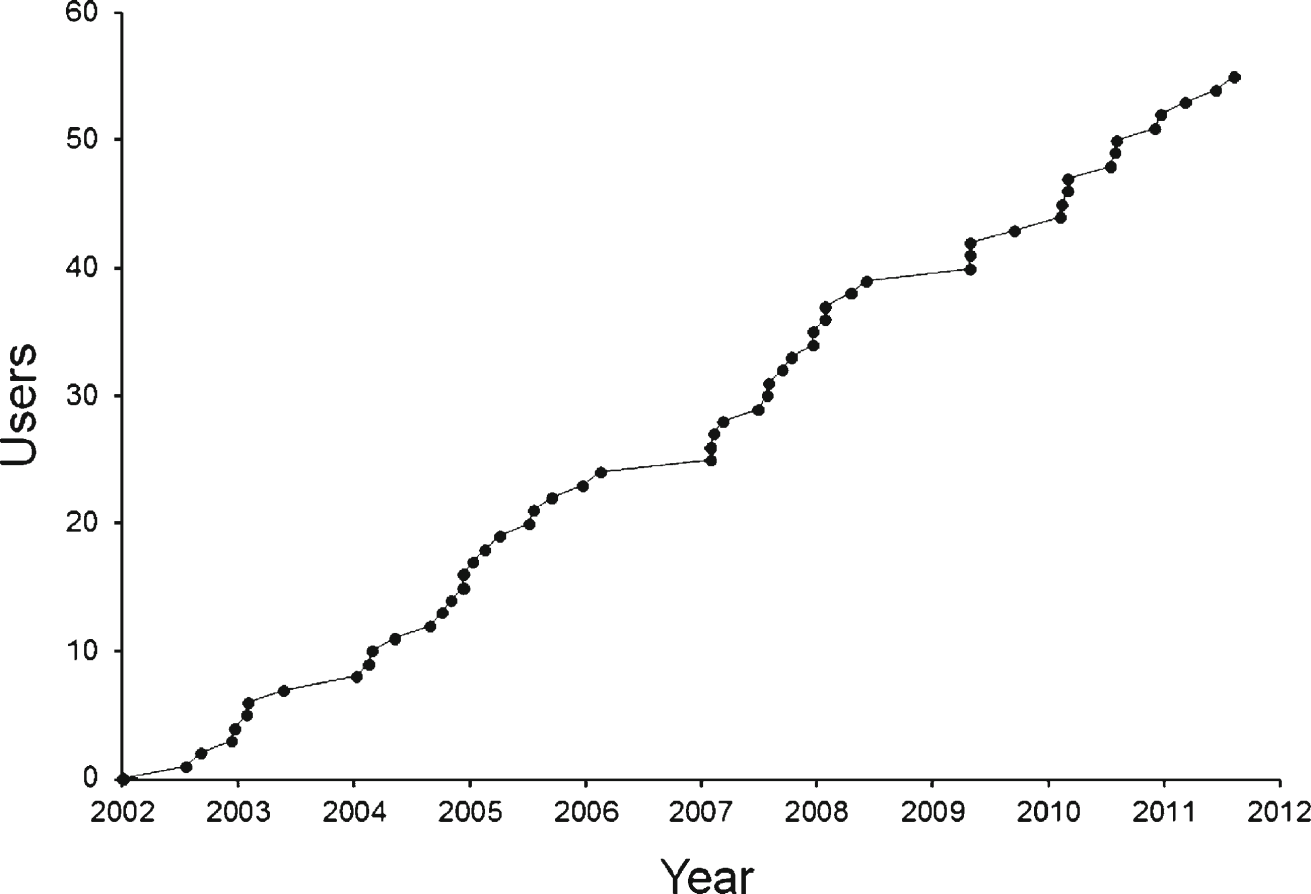
Cumulative number of UK users of TOPS since its launch in 2002

**Fig. 2:**
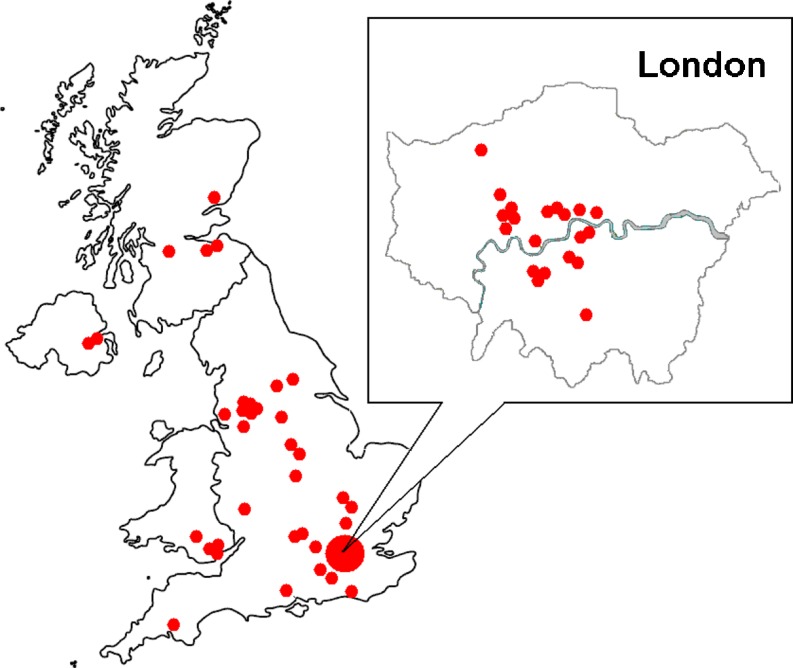
Locations of TOPS users

Since we launched TOPS in 2002, 55 units (Fig. 1) have registered to use it: 29 CROs, 5 pharmaceutical companies, 13 universities and 8 hospitals, throughout the UK (Fig. 2). All UK commercial and many non-commercial units have registered to use TOPS. Users entered 124,806 subjects in TOPS between 2002 and mid-2011. CROs are the biggest users, contributing 116,843 (93.6%) of all entries.

Table 2 summarises our experience of over-volunteering in the period 1997–2011. In the first 2 years after we started using TOPS, potential over-volunteers increased from about 1% to a peak of 4.6%. Thereafter, there was a progressive fall, and in the last few years the incidence has been <1%. Table 3 shows that we now find most potential over-volunteers by using TOPS.

**Table 2:** Number of healthy subjects who came for screening at our unit, and the number and percentage of these who attempted to volunteer for a trial within 3, 2 or 1 months of completing a previous trial

Year	Number of volunteers who came for screening	Number (%) of volunteers completing a trial elsewhere within the previous:		
		3 months	2 months	1 month
1997–2001	6,998	68 (1.0)	56 (0.8)	31 (0.4)
2002^a^	1,490	52 (3.5)	43 (2.9)	22 (1.5)
2003	1,811	83 (4.6)	62 (3.4)	23 (1.3)
2004	1,092	18 (1.6)	15 (1.4)	9 (0.8)
2005	836	20 (2.4)	14 (1.7)	8 (1.0)
2006	1,583	38 (2.4)	27 (1.7)	14 (0.9)
2007	1,257	20 (1.6)	14 (1.1)	3 (0.2)
2008	1,349	13 (1.0)	11 (0.8)	5 (0.4)
2009	767	5 (0.7)	5 (0.7)	3 (0.4)
2010	1,114	9 (0.8)	9 (0.8)	6 (0.5)
2011^b^	990	5 (0.5)	5 (0.5)	2 (0.2)

^a^TOPS launched in 2002

^b^First half of year only

**Table 3:** Number and methods of detecting healthy subjects who attempted to volunteer for a trial in our unit within 3 months of completing a previous trial

Methods	Year										
	1997–2001	2002	2003	2004	2005	2006	2007	2008	2009	2010	2011^a^
TOPS^b^	–	–	37	12	17	30	14	13	3	8	5
Signs^c^	0	13	2	4	0	0	0	0	0	0	0
We called other CROs	34	18	11	1	1	1	1	0	0	0	0
Other CROs called us	15	19	22	1	0	1	1	0	1	0	0
Volunteer admitted when challenged	7	1	3	0	1	1	1	0	0	1	0
GP replies	12	1	8	0	1	5	3	0	1	0	0
Total	68	52	83	18	20	38	20	13	5	9	5^a^

CRO, Contract research organisation; GP, general practitioner

^a^First half of year only

^b^TOPS was launched in 2002; no potential over-volunteer was identified by TOPS in that year

^c^Venepuncture marks on forearm, electrocardiogram electrode marks (or electrodes still in place) or haematology results

Table 2 also shows by how much the incidence of potential over-volunteers in our unit is reduced if over-volunteering is defined as <1 month or <2 months between trials. An interval of <2 months reduces the incidence only slightly, while an interval of <1 month reduces the incidence substantially, to 0.2–0.5% in recent years.

We have other examples of the effectiveness of TOPS. Several subjects have volunteered for trials in our unit, but TOPS showed that they were still taking part in trials elsewhere. Some GPs have informed us that their patients had not taken part in a trial in the previous 3 months, but TOPS showed that they had. On the other hand, some GPs have informed us that their patients had taken part in a trial in the previous 3 months, whereas TOPS showed that they had been registered but never dosed, so we could recruit them. The reasons why they had not been dosed included not meeting the protocol selection criteria or cancellation of the studies.

## Discussion

We were surprised by the increase in our detection of over-volunteering during the first 2 years after the introduction of TOPS and attribute this increase to the efficacy of TOPS compared with our former methods of detection. Almost certainly, TOPS helped identify potential over-volunteers whom we would previously have missed. After the initial surge, the incidence of over-volunteering fell. As the uptake of TOPS increased, it not only helped us to identify potential over-volunteers, but also acted as a deterrent as volunteers became aware of its efficiency. Other units have had similar experiences.

Currently, only a few subjects attempt to volunteer for our studies within <3 months after completing one elsewhere. Adopting a 2-month interval, which would probably be enough for most studies of small molecules, reduces the calculated incidence only slightly. A 1-month interval, as recommended by the FDA [3], reduces the incidence substantially, but most UK investigators would agree that 1 month is insufficient.

As far as we know, no volunteer has ever participated in one of our trials while taking part in another elsewhere. However, a few subjects have succeeded in starting a trial in our unit within 28 days after the last dose in trials in other units. Those units had just started to use TOPS, but were not doing so consistently, otherwise TOPS would have identified the subjects as potential over-volunteers.

Not all attempts to over-volunteer are intentional. Some subjects genuinely misunderstand instructions about not leaving too short an interval between trials, or overestimate the interval since their last study.

We have been surprised by some replies from GPs to our requests for information about their patients. Occasionally GPs tell us that the patient has not done a trial within the previous 3 months, when TOPS shows that they have (*false negative*), and occasionally GPs tell us that the patient has done a trial within the previous 3 months, when TOPS shows that they were screened but never dosed (*false positive*). We have several examples of each, which clearly demonstrate that a reply from the GP alone is not sufficient to prevent over-volunteering and that it can falsely incriminate a volunteer.

The success of TOPS depends upon users registering every volunteer and cooperating with each other when over-volunteering is suspected. In our experience, cooperation has been good.

Two systems with aims similar to TOPS are used in some other EU countries.

1. VIP (Volunteer Inclusion Period) Check [11] is privately owned system based in Germany. Users enter the volunteer’s full name, date of birth, sex, nationality, start and end dates of the study and whether it involves exposure to radioactivity. Users pay fees that depend on usage. VIP Check reports a volunteer’s participation in another study for 60 days before and 60 days after completing one, as well as participation in more than one study at a time. It is currently used by 22 units (17 CROs, 2 pharmaceutical companies and 3 university units) in Germany, Belgium, Netherlands and Switzerland [12]. Only 40,000 volunteers have been entered in VIP Check since 2000 [12], which would suggest that it is not used regularly. No information is available about whether VIP Check prevents over-volunteering.
2. VRB (Volontaires pour la Recherche Biomédicale) [13] is a French national database run by the Ministry of Health. It is web-based, mandatory to use according to French law [14] and free to users. Users must enter the volunteer’s Social Security number and initials, the start and end dates of the study and payment. The maximum that a volunteer can earn in a year is €4,500, and there is a post-study exclusion period. There is no published information about the usage of VRB or whether it prevents over-volunteering.

A recent report claimed that healthy subjects travel to Belgium from nearby EU countries to take part in phase 1 trials (‘tourist volunteers’). This report led to a call for a single mandatory system to prevent over-volunteering throughout the EU [12]. We have no evidence that healthy subjects travel from other EU countries just to volunteer for a clinical trial in our unit. However, unlike NINO, a passport number is not for life, so if we have any concerns about the validity of a non-UK volunteer, our policy is not to recruit them. Cultural differences on storing personal information would make it difficult to implement a single EU system. Funding would be needed to set up, maintain and run the system. TOPS is a low-cost system that is free to all users, and it serves the UK well; as such, there seems little reason to change it. The UK is the only country in the world with a widely used system to prevent over-volunteering.

Could TOPS be improved? A biometric identifier would improve TOPS but, although technology has advanced and costs have reduced since we set up TOPS, costs would still limit its use substantially. TOPS usage could be extended to other countries, particularly those in which citizens have identity cards. Identity cards are the norm in many EU countries, and in some, such as Belgium [15], people must carry their identity cards at all times.

Since TOPS was launched, UK regulations [16, 17] have required investigators to have procedures to prevent over-volunteering. TOPS helps units to comply with these. A recent UK bioethics report called for mandatory use of TOPS [18].

In conclusion, only a few healthy subjects now attempt to over-volunteer in our unit, and to the best of our knowledge very few succeed. TOPS not only helps to prevent over-volunteering, but also deters subjects from trying to do so. TOPS makes recruiting volunteers more efficient, prevents unnecessary screening of volunteers, and helps units to comply with UK regulations. Feedback from other units is required to determine whether our findings apply to UK units in general.
